# Sleep quality in cigarette smokers and nonsmokers: findings from the general population in central China

**DOI:** 10.1186/s12889-019-6929-4

**Published:** 2019-06-24

**Authors:** Yanhui Liao, Liqin Xie, Xiaogang Chen, Brian C. Kelly, Chang Qi, Chen Pan, Mei Yang, Wei Hao, Tieqiao Liu, Jinsong Tang

**Affiliations:** 1 Department of Psychiatry, Sir Run Run Shaw Hospital, Zhejiang University School of Medicine, Hangzhou, Zhejiang China; 2 Key Laboratory of Medical Neurobiology of Zhejiang Province, Hangzhou, Zhejiang China; 3 Department of Psychiatry, the Second Xiangya Hospital, Central South University, Changsha, 410011 Hunan China; 4 National Clinical Research Center on Mental Disorders, Changsha, 410011 Hunan China; 5 Hunan Key Laboratory of Psychiatry and Mental Health, Changsha, 410011 Hunan China; 6 Changsha Social Work College, 22 Xiangzhang Rd, Changsha, 410116 Hunan China; 7 Department of Sociology & Center for Research on Young People’s Health (CRYPH), Purdue University, 700 W State Street, West Lafayette, IN 47907 USA; 8 Clinical Psychology Department, the Third Xiangya Hospital, Central South University, Changsha, Hunan 410013 People’s Republic of China; 9 Shenzhen Mental Health Center, Shenzhen Kangning Hospital, Shenzhen, 518020 People’s Republic of China

**Keywords:** Sleep quality, Sleep disturbances, Cigarette smoking

## Abstract

**Background:**

Sleep problems are common in the general population. Cigarette smoking is common in the general population of China. Examinations of the prevalence of poor sleep quality among Chinese smokers and nonsmokers are still lacking. This study was designed to examine sleep quality and sleep disturbances among cigarette smokers and nonsmokers in the general population in central China.

**Methods:**

In this population-based sampling project, we used a multi-stage sampling method to recruit survey participants from September 2012 to October 2012 in rural and urban areas of Hunan province, China. A total of 27,300 subjects were sampled from the general population and 26,282 completed the self-report of cigarette smoking characteristics. Cigarette smoker was defined as having smoked ≥100 cigarette in a lifetime and smoked during the last 28 days. Cigarette smoking characteristics were obtained from smokers, including cigarettes per day, years of smoking, quit attempts, and smoking cravings. The Pittsburgh Sleep Quality Index (PSQI) was applied to assess quality of sleep and sleep disturbances (PSQI score > 5).

**Results:**

Significantly more smokers than nonsmokers demonstrated poor sleep quality and sleep disturbances. Among smokers, linear regression analyses showed that poor sleep was inversely associated with cigarettes per day, and positively associated with years of smoking, quit attempts, and smoking craving. Logistic regression analysis showed that quit attempts and smoking cravings were associated with higher odds of sleep disturbances.

**Conclusions:**

Sleep disturbances were more prevalent among cigarette smokers than nonsmokers. Smokers also varied in sleep problems on the basis of the characteristics of their smoking. Smokers should be informed about the link between cigarette smoking and poor sleep quality, and should be advised that one of several important health benefits from smoking cessation could be the improvement of sleep quality. Sleep therapy should be recommended as an adjunctive treatment for smoking cessation.

## Background

China is the biggest consumer of cigarettes in the world [1]. Cigarette smoking remains a serious health burden, and contributes to a significant proportion of morbidity and mortality throughout the world. In 2015, there were 933.1 million daily smokers in the world, and 6.4 million deaths (11.5% of global deaths) were attributable to cigarette smoking worldwide. Over three-quarters of deaths attributable to smoking were among men, and 52.2% of these deaths took place in only four countries (China, India, United States, and Russia) [2]. Cigarette smoking is the primary causal factor for more than 30% of all cancer deaths, for approximately 80% of deaths from COPD, and for a significant proportion of early cardiovascular disease and related deaths [3]. Yet, it may also contribute indirectly to other health problems (such as physical or mental distress) through impairing sleep among smokers [4, 5].

Nicotine, an addictive stimulant agent in cigarettes, not only makes smoking cessation difficult but also causes withdrawal symptoms, including a nightly withdrawal symptom related to poor sleep, or even insomnia [6, 7]. Poor sleep is of growing concern to global public health officials because it is associated with impairments in emotional and cognitive performance [8, 9], impaired quality of life [10], non-suicidal self-injury [11], as well as increased risk for numerous physical medical conditions, such as obesity, cardiovascular disease, and related mortality [12, 13].

A line of research has explored the association between cigarette smoking and poor sleep. Several studies indicated poorer sleep quality among smokers [4, 14–16], with some showing variation in sleep disturbances by smoking characteristics, such as light versus heavy smoking [17–19]. Besides smoking’s potential impact on sleep quality, there has also been some research suggesting that sleep quality, in turn, may affect smoking behaviors [16, 20]. A line of evidence indicates that smokers with poor sleep quality were significantly less likely to quit smoking than those without sleep disturbances [21, 22]. In this regard, the relationship of smoking to sleep quality may be complex and bi-directional, but this highlights the durability of the relationship between smoking and sleep health.

In China, sleep problems have been well documented in a wide range of subpopulations, including the elderly [23, 24], younger adults [25], adolescents [26, 27], children [28, 29], drug users [30], and patients with Parkinson’s disease [31]. However, few studies have examined the prevalence of poor sleep quality or sleep disturbances among Chinese smokers and nonsmokers. A survey of 3285 Changchun city residents showed that poor sleep quality was associated with cigarette smoking [32]. However, a cross-sectional survey of 3289 middle-aged and elderly Chinese did not find significant association between sleep quality and smoking habits [33]. To extend research in this area study, we examined the prevalence of cigarette smoking in a general population sample in central China, compared sleep disturbances between cigarette smokers and nonsmokers, and evaluated smoking characteristics among smokers that may be associated with sleep problems.

## Method

### Participants and procedure

The study participants and procedure have been previously presented in detail [34]. Data were collected from September 2012 to October 2012. A community-based method was used to select participants (12 years of age or older) in the general population from Hunan Province of China. A population-based cross-sectional survey design was used to explore the prevalence of cigarette smoking, and the prevalence of sleep disturbances in cigarette smokers and nonsmokers. We randomly sampled locations from all divisions (13 prefecture level cities and 1 autonomous prefecture) of Hunan province. There were 135 sites in total, which include 93 urban street districts (31 capital cities, 23 prefecture level cities, 23 county level cities, 16 small towns) and 42 rural villages. In each prefecture level city or autonomous prefecture, at least 1 prefecture level city, 1 county level city, 1 small town and 1 rural village was selected. Persons aged 12 years or older and living in their current residence for 5 years or longer were randomly selected from each household without replacement. A sample of 27,300 people were randomly sampled from the selected sites and invited to participate in the study, with completing self-report cigarette smoking characteristics as one of the inclusion criteria. Thus, after the exclusion of missing demographic and smoking information, 26,282 participants were available for the final analysis. The ages of the participants ranged from twelve to ninety-nine years, with an average age of thirty-eight years.

Inclusion Criteria:

- Aged 12 years or older
- Living in their current residence for 5 years or longer
- Be able to recognize 1500–2000 Chinese characters (fully recognize the Instructions)
- Willingness to participant the survey

Exclusion Criteria:

- Less than 12 years old
- Failure to provide informed consent or unwilling to participant

### Procedure

At each selected site, participants reached door to door were invited to fill out the survey if they consented to participate. Every household member aged 12 or more years was encouraged to participate via a face-to-face interview. The interviewers were medical students or psychiatrists who received a 2-h training regarding the objectives and procedures of the survey. On average, it took approximately 20 min to complete the survey. As part of the consent process, all participants were provided with a detailed explanation of the aims of the study and study expectations. Participants were advised of their ability to withdraw from the study at any point. Issues of confidentiality and anonymity were discussed. All participants were encouraged to answer the questionnaire independently and as soon as possible. Those who were unable to recognize 1500–2000 Chinese characters (cannot fully recognize the Instructions) were defined as illiterate. For those participants, the staff would read and explain each question, and helped illiterate participants to write down their answers if necessary.

### Measures

Cigarette smoking status: cigarette smoker was defined as having smoked more than one hundred cigarettes during the subject’s lifetime and smoked during the last 28 days [35]. Cigarette smoking characteristics were obtained from smokers, including cigarettes per day, years of smoking, number of quit attempts, and smoking craving assessed by The Visual Analogue Scale for Craving (VASc) [36, 37] with scores ranging from 0 (no craving) to 10 (the strongest craving). The VASc is a 10-cm line with “no craving at all” on the left and “the strongest craving” on the right. Participants were asked to rate their craving for smoking at present on the VASc. Alcohol drinking was defined as drunk > 30 g alcohol (equal to 900 ml beer) per week. Body Mass Index (BMI) was obtained from self-reported height. It was calculated as weight (in kilograms) divided by height (in meters) squared. Sleep quality was measured by The Pittsburgh Sleep Quality Index (PSQI) [38, 39] with scores ranging from 0 and 21 points. The PSQI measures multiple dimensions of sleep quality. Higher scores reflect poorer sleep quality. As the commonly established cutoff is a score greater than 5, an indicator of sleep disturbances or insomnia was defined as a dichotomous variable with PSQI score more than five. Covariates included age, gender, Body Mass Index (BMI), marital status, employment status, and residential locale.

### Analysis

Statistical analysis was performed using the SPSS (Version 22, SPSS Inc., Chicago, IL, USA). Descriptive statistics were examined for demographic and sleep quality characteristics. The prevalence of sleep disturbances (poor sleepers) among overall participants, between smokers and nonsmokers was examined in different age stages, levels of education, marital status and living regions. Differences in percentage or mean differences analyzed between nonsmokers and smokers via Chi-squared tests and the Wilcoxon rank sum test (Mann-Whitney U-test due to the non-normal data distribution), respectively. Linear and logistic regression models were used to examine the associations between smoking characteristics and sleep quality and sleep disturbances, respectively among the smokers. Smoking characteristics included cigarettes per day, years of smoking, number of quit attempts, and smoking cravings, while controlling for age, gender, BMI, employment status, marital status, and rural or urban residence. Age and BMI treated as categorical factors and others as categorical factors. The significance level was set at *p*-value threshold of 0.05.

## Results

A total of 27,300 subjects were sampled from the general population and 26,282 (97.3% response rate) completed the self-report of cigarette smoking characteristics. The overall prevalence of cigarette smoking was 25.3%, with 45% smokers in male population and 3% smokers in female population. In the male general population, the prevalence of smoking was 6.6% in adolescents 12 to 17 years old, 36.9% between age 18 to 29 years old, 50.7% between age 30 to 39 years old, 57.9% between age 40 to 49 years old, 57.4% between age 50 to 59 years old and 51.1% in adults aged 60 years or older. In the female general population, the prevalence of smoking was 0.2% in adolescents 12 to 17 years old, 2.6% between age 18 to 29 years old, 2.4% between age 30 to 39 years old, 3.3% between age 40 to 49 years old, 3.7% between age 50 to 59 years old and 5.1% in adults aged 60 years or older.

Compared with nonsmokers, smoking participants were older, had a lower level of education, higher Body Mass Index (BMI), higher personal income, as well as were more likely to be employed, married, urban residents, and overwhelmingly more likely to consume alcohol. Detailed demographic characteristics of the entire sample, nonsmokers and smokers are shown in Table 1.

**Table 1:** Demographic and smoking characteristics in overall participants, nonsmokers, and cigarette smokers

Variables	Overall	nonsmokers	smokers	p*
	(*n* = 26,282)	(*n* = 19,632)	(*n* = 6650)	
Age (yrs), M ± SD	38.1 ± 16.06	36.6 ± 16.26	42.2 ± 14.57	< 0.001^*^
Range (yrs)	12–99	12–99	12–93	
12–17, %	10.2%	13.2%	1.7%	–
18–29, %	23.3%	24.5%	19.2%	–
30–39, %	17.2%	17.3%	17.0%	–
40–49, %	28.8%	26.3%	36.0%	–
50–59, %	9.6%	8.3%	13.1%	–
≥60, %	11.0%	10.3%	13.0%	–
Education (yrs), M ± SD	10.6 ± 3.79	10.7 ± 3.81	10.4 ± 3.70	< 0.001^*^
Illiteracy or Primary school%	19.0%	18.6%	20.2%	–
Middle school%	32.5%	32.4%	32.8%	–
High school%	23.3%	22.9%	24.8%	–
University%	25.2%	26.1%	22.3%	–
BMIˣ, M ± SD	21.2 ± 2.91	21.0 ± 2.92	21.9 ± 2.76	< 0.001
Personal income (CNY)	2228 ± 3388.98	2015.5 ± 3227.15	2818.0 ± 3738.50	< 0.001
Employed %	78.1%	73.3%	92.4%	< 0.001
Female %	46.8%	60.9%	5.5%	< 0.001
Married %	67.9%	64.8%	77.0%	< 0.001
First marriage%	67.3%	64.3%	76.1%	–
Re-married %	0.6%	0.5%	0.9%	–
Unmarried %	32.1%	35.2%	23.0%	< 0.001
Never married%	26.4%	29.9%	16.1%	–
Divorced %	1.9%	1.7%	2.6%	–
Widowed %	3.2%	3.1%	3.5%	–
Separated %	0.5%	0.4%	0.8%	–
Rural region%	31.2%	30.6%	33.1%	< 0.001
Urban region %	68.8%	69.4%	66.9%	< 0.001
Provincial capital city%	22.8%	23.5%	21.1%	–
Prefecture-level city%	16.4%	16.5%	15.9%	–
County-level city%	16.9%	16.7%	17.6%	–
Small town%	12.5%	12.6%	12.4%	–
Smoking years	**–**	**–**	16.7 ± 12.17	**–**
Smoked cigarettes/day	**–**	**–**	18.4 ± 14.84	**–**
Quit attempts response (yes)%	**–**	**–**	28.3%	–
Attempts	**–**	**–**	2.9 ± 6.32	**–**
Smoking craving◊	**–**	**–**	4.9 ± 2.42	**–**
Alcohol drinker%	20%	8.2%	54.8%	< 0.001
Drinking years	15.5 ± 12.58	13.6 ± 12.48	16.3 ± 12.53	< 0.001
Alcoholic drinks per week	4.4 ± 4.31	4.0 ± 4.01	4.5 ± 4.43	< 0.001

*M* mean, *SD* standard deviation, *n* number; %: the percentage of subjects

Cigarette smoker was defined as smoked > 100 cigarettes in the life time

Alcohol drinking was defined as drunk > 30 g alcohol (equal to 900 ml beer) per week

Difference in percentage or mean differences analyzed between nonsmokers and smokers by the Chi-squared test and the Wilcoxon rank sum test (Mann-Whitney U-test) respectively

* Significant difference between groups of nonsmokers and smokers, *p* < 0.01

% The distribution of participants in each group or subgroup

ˣ Body Mass Index (BMI) was obtained from self-reported height. It was calculated as weight (in kilograms) divided by height (in meters) squared

◊ Smoking craving was measured by the Visual Analogue Scale for Craving (VASc) using a scale from 0 to 10, where a score of 0 represents null craving and a score of 10 represents the most extreme craving

Significantly more smokers than nonsmokers demonstrated poor sleep quality across a range of sleep dimensions as well as sleep disturbances (via a PSQI global score of over 5), see Table 2. Smokers exhibited poorer scores than nonsmokers on subjective sleep quality (0.91 vs 0.79), sleep latency (0.82 vs 0.77), sleep duration (0.67 vs 0.55), habitual sleep efficiency (0.35 vs 0.32), and sleep disturbances (0.86 vs 0.75). Smokers also exhibited greater daytime dysfunction (1.00 vs .084) and a greater proportion reported sleep disturbances (32.7% vs 24.5%). Notably, the worse scores on sleep quality for smokers spanned the life course. Sleep quality was also worse for smokers compared with nonsmokers despite accounting for differences in educational levels, marital status and living regions (Table 3). As detailed in Table 3, a greater proportion of cigarette smokers reported sleep disturbances across all life course stages, with a very noticeable gap among adolescents 12 to 17 years old.

**Table 2:** Score of the PSQI components and PSQI total score in overall participants, nonsmokers, and cigarette smokers

Variables	Total	Nonsmokers	Smokers	P*
	(*n* = 26,035)	(*n* = 19,466)	(*n* = 6569)	
Subjective sleep quality, M ± SD	0.82 ± 0.65	0.79 ± 0.64	0.91 ± 0.66	< 0.001
Sleep latency, M ± SD	0.77 ± 0.73	0.75 ± 0.72	0.82 ± 0.75	< 0.001
≤15 min, %	38.6%	39.3%	36.4%	–
16-30 min, %	48.2%	48.1%	48.2%	–
31-60 min, %	11.0%	10.4%	12.6%	–
> 60 min, %	2.3%	2.1%	2.8%	–
Sleep duration, M ± SD	0.58 ± 0.78	0.55 ± 0.76	0.67 ± 0.81	< 0.001
> 7 h, %	58.1%	59.9%	52.6%	–
5–7 h, %	40.4%	38.7%	45.6%	–
< 5 h, %	1.5%	1.4%	1.8%	–
Habitual sleep efficiency*, M ± SD	0.33 ± 0.73	0.32 ± 0.71	0.35 ± 0.76	0.047
≥85%, %	78.6%	78.9%	77.9%	–
75–84%, %	13.3%	13.3%	13.2%	–
65–74%, %	4.6%	4.5%	4.7%	–
< 65%, %	3.6%	3.4%	4.2%	–
Sleep disturbance, M ± SD	0.78 ± 0.55	0.75 ± 0.56	0.86 ± 0.52	< 0.001
Need for sleep medications, M ± SD	0.11 ± 0.38	0.11 ± 0.37	0.12 ± 0.42	0.807
Not during the past month, %	91.4%	91.4%	91.3%	–
Less than once a week, %	6.9%	7.1%	6.3%	–
Once or twice a week, %	1.4%	1.3%	1.8%	–
≥3 times a week, %	0.4%	0.3%	0.6%	–
Daytime dysfunction, M ± SD	0.88 ± 0.84	0.84 ± 0.83	1.00 ± 0.86	< 0.001
PSQI total score, M ± SD	4.26 ± 2.67	4.11 ± 2.62	4.72 ± 2.75	< 0.001
sleep disturbances (PSQI > 5), %	26.5%	24.5%	32.7%	< 0.001

*M* mean, *SD* standard deviation, *n* number, % the percentage of subjects, *PSQI* Pittsburgh Sleep Quality Index

Difference in percentage or mean differences analyzed between nonsmokers and smokers by the Chi-squared test and the Wilcoxon rank sum test (Mann-Whitney U-test) respectively

* Significant difference between groups of nonsmokers and smokers, *p* < 0.01

The score range is 0–70, with higher scores indicating poorer functioning

Habitual sleep efficiency^*^ = total hours of sleep/(get-up time − bedtime) × 100%

**Table 3:** Percentage of sleep disturbances (poor sleepers) in different age stages, levels of education, marital status and regions among overall participants, nonsmokers, and cigarette smokers

Variables	insomnia (PSQI > 5), %			
	Overall	nonsmokers	smokers	p*
Age (yrs)				
12–17, %	15.4%	14.6%	33.3%	< 0.001
18–29, %	24.7%	23.3%	30.0%	< 0.001
30–39, %	23.6%	21.4%	30.3%	< 0.001
40–49, %	25.0%	23.5%	28.2%	< 0.001
50–59, %	31.8%	30.0%	35.1%	0.011
≥60, %	45.2%	43.4%	49.5%	0.003
Education (yrs)				
Illiteracy or Primary school%	32.6%	31.4%	35.9%	0.003
Middle school%	22.4%	20.3%	28.6%	< 0.001
High school%	27.0%	24.4%	34.3%	0.011
University%	26.6%	24.6%	33.5%	0.003
Marital status				
Married %	26.0%	25.0%	31.2%	< 0.001
First marriage%	26.7%	25.0%	31.0%	–
Re-married %	39.6%	35.5%	46.4%	–
Unmarried %	26.6%	24.1%	37.7%	< 0.001
Never married%	22.0%	20.1%	32.5%	–
Divorced %	38.5%	39.6%	41.9%	–
Widowed %	54.2%	53.0%	57.5%	–
Separated %	35.8%	32.9%	42.0%	–
Regions				
Rural region%	29.4%	27.3%	34.6%	< 0.001
Urban region %	25.5%	23.5%	31.6%	< 0.001
Provincial capital city%	27.0%	25.1%	33.5%	–
Prefecture-level city%	23.5%	21.3%	30.0%	–
County-level city%	25.9%	23.9%	31.3%	–
Small town%	24.8%	22.9%	30.7%	–

%: the percentage of subjects. *PSQI* Pittsburgh Sleep Quality Index

* Significant difference between groups of nonsmokers and smokers, *p* < 0.01

Percentage differences were analyzed between nonsmokers and smokers by the Chi-squared test

Among the smokers, we explored the association between sleep quality, sleep disturbances and smoking characteristics, including cigarettes per day, years of smoking, number of quit attempts, and smoking cravings, while controlling for age, gender, BMI, employment status, marital status, and rural or urban residence (see Table 4). Linear regression analyses showed that poor sleep was inversely associated with cigarettes per day (*b* = − 0.004). Poor sleep was positively associated with years of smoking (*b* = − 0.004), quit attempts (*b* = − 0.004), and smoking cravings (*b* = − 0.004). Logistic regression analysis showed that higher odds of sleep disturbances was associated with the number of quit attempts (OR = 1.100) and smoking craving (OR = 1.107).

**Table 4:** Predictors of sleep quality (PSQI total score) and sleep disturbances (PSQI > 5) in cigarette smokers

	PSQI	Insomnia
	*B* (SE)	OR (95% CI)
Cigarettes per day	−0.004 (0.002) ^**^	0.998 (0.994, 1.002)
Years Smoking	0.013 (0.004) ^**^	1.005 (0.999, 1.012)
Quit Attempts	0.127 (0.019) ^***^	1.100 (1.066, 1.135) ^***^
Smoking Craving	0.150 (0.017) ^***^	1.107 (1.077, 1.137) ^***^

*** *p* < .001; ** *p* < .01

*B* the unstandardized coefficients, *SE* standard error, *OR* odds ratios, *CI* 95% confidence intervals, *BMI* Body Mass Index

## Discussion

To our knowledge, this investigation is the first study to examine broadly the relationship between cigarette smoking and sleep quality in a Chinese general population. In comparison to nonsmokers, smokers demonstrated poorer sleep quality and a greater proportion of smokers reported sleep disturbances. These results are consistent with findings from prior studies in other populations [4, 14–16] that smokers had poorer sleep quality than nonsmokers.

Notably, the results presented suggest that cigarette smoking was associated with numerous dimensions of sleep quality – subjective sleep quality, sleep latency, sleep duration, habitual sleep efficiency, and sleep disturbances – rather than related to a single dimension of sleep quality. As such, the disruption of sleep quality among smokers may not be occurring via a single mechanism. Rather, smoking may affect sleep in diverse ways. It may directly relate to the active ingredient, nicotine. First of all, the physiological desire for additional nicotine during sleep may cause smokers to awaken, leading to insomnia. Secondly, nicotine itself is a stimulant that increases alertness [40], and the use of it too close to bedtime may affect sleep latency. It also affects quality of sleep by increasing the risk for snoring and obstructive sleep apnea [41] and disrupting the circadian clock [42].

A greater proportion of smokers reported sleep disturbances across all different age stages, which suggests that these processes are unfolding across the life course. Similar results shown from Thai college students, compared to those who reported never smoking, current smokers were less likely to report short sleep duration, more likely to have long sleep, daytime dysfunction due to poor sleep, and to use sleep medicines [43]. Also, more smokers reported poor sleep quality than nonsmokers did despite of the differences in educational levels, marital status and living regions.

Among smokers, we identified that greater cigarette consumption was actually associated with better sleep quality among smokers. Our research is not the first to encounter such a counter-intuitive findings. Riedel and colleagues identified that smokers who smoked less than 15 cigarettes per day reported reduced total sleep time, while heavier smokers failed to show a similar association with insomnia [17]. We may speculate that lighter smokers may consume tobacco at more erratic intervals throughout the day, while heavy smokers consume tobacco on a more consistent schedule. Thus, the variation in nicotine administration over time may play a role in how sleep quality unfolds at night.

By contrast, the number of years smoking, the number of quit attempts, and intensity of smoking cravings were all positively associated with poorer sleep quality among smokers. The longer duration of smoking may shape how nicotine administration shapes physiological processes in a manner accumulates over time. A mice study found that both chronic and acute exposure to cigarette smoke can alter the expression of clock genes, and the natural circadian clocks were disrupted with increased tobacco exposure [42]. The number of quit attempts may signal dissatisfaction with side effects, such as poor sleep quality, attributable to cigarette smoking, and indicates a need for support for individuals attempting smoking cessation in a manner that promotes sleep health. Poor sleep quality often is a risk factor for relapse following quit attempts in addictive substance users [44], and was often associated with quitting self-efficacy [45]. Thus, sleep therapy was recommended as an adjunctive treatment for smoking cessation [46]. Lastly, smoking cravings may highlight differences in physiological response to nicotine, as well as highlight the manner in which sleep disruption may be a component of withdrawal processes. Especially those with symptoms of “nocturnal sleep-disturbing nicotine craving”, which characterized by craving for cigarettes during the individual sleep times [47].

### Limitations

Although the findings from this study relate to sleep quality and sleep disturbances or insomnia, we only used self-reported PSQI for these assessments rather than objective measure, such as a polysomnogram. Findings that replicate in a sample with both subjective and objective sleep assessments, such as the administration of Diagnostic and Statistical Manual of Mental Disorders (DSM–5) criteria for insomnia and polysomnography would strengthen these results. Nonetheless, the Chinese Version of the Pittsburgh Sleep Quality Index (PSQI) is a psychometrically sound and well-validated measure of sleep quality and disturbance for community-based studies of primary insomnia [39]. Another limitation with this study is the lack of control over potentially unobserved confounding variables. For example, we did not assess anxiety and depressive symptoms, which can negatively impact sleep and may account for the perceived relation between smoking and sleep disturbances [22]. Also, the cross-sectional nature of this survey inhibits causal inference and clarity on the directionality of the associations reported within the study. Placed within the context of the wider literature, these results provide additional evidence suggesting the disruption of sleep associated with cigarette smoking among Chinese citizens. Additionally, within this study, we do not have the data about the population in different age periods within all geographic divisions (13 prefecture level cities and 1 autonomous prefecture) of Hunan province. Thus, we cannot apply sampling weights or otherwise make prevalence adjustments. However, the population characteristics of all 14 divisions are otherwise similar and the sample approximates the demographic makeup of this region. We nonetheless acknowledge the lack of sampling weights as a limitation. Lastly, the percentage of female smokers was extremely low, but this corresponds to a strong gender gap in smoking within China and is similar to such stark differences found in other studies of smoking in China [1].

Future research should explore the mechanisms of associations between smoking duration, quit attempts, and smoking cravings with poorer sleep quality in cigarette smokers, as well as explore the mechanisms by which smoking and sleep problems may be influenced by other factors, such as lifestyle choices or environmental influences. To better understand how intervening on one behavior can improve another behavior, smoking cessation interventions should include sleep problems as one of secondary outcomes, and vice versa.

## Conclusions

These findings suggest that sleep disturbances were more prevalent among cigarette smokers than nonsmokers in China, although the prevalence of cigarette smoking was predominantly among men (although commonly women have poorer sleep quality than men do [48]). Individuals who are cigarette smokers should be informed about the links between cigarette smoking and poor sleep quality, and they should be advised that one of a range of important health benefits from smoking cessation could be the improvement of sleep quality. Sleep therapy should be recommended as an adjunctive treatment for smoking cessation.

## Abbreviations

BMI: Body Mass Index
PSQI: The Pittsburgh Sleep Quality Index
VASc: Visual Analogue Scale for Craving
